# Two major human phenotypes of MICA molecules and their differential activation to NK cells via NKG2D receptor

**DOI:** 10.3389/fimmu.2025.1563872

**Published:** 2025-05-19

**Authors:** Qizhi Luo, Xiangli Yin, Quan Zhu, Weiguang Luo, Rongjiao Liu, Leiyan Wei, Yizhou Zou

**Affiliations:** Department of Immunology, School of Xiangya Basic Medical Sciences, Central South University, Changsha, China

**Keywords:** MICA polymorphisms, NKG2D receptor, NK cell, binding affinity, immune regulation

## Abstract

**Introduction:**

The major histocompatibility complex class I-related gene A (*MICA*), is a highly polymorphic gene, serve as a crucial role in immune regulator through its interaction with the NKG2D receptor on natural killer (NK) cells. These polymorphisms may influence immune responses, disease susceptibility, and transplant outcomes. However, the precise mechanisms by which *MICA* polymorphisms modulate NKG2D receptor activation remain poorly understood.

**Methods:**

We analyzed 29 representative MICA polymorphic molecules that cover the most prevalent alleles in the population. These variants were systematically examined through Luminex bead assays, monoclonal antibody binding studies, and NKG2D-Ig fusion protein assays. NKG2D receptor activation was assessed *in vitro* using NKG2D reporter cells, while NK cell-mediated cytotoxicity was evaluated through NKL cell killing assays against target cells expressing either Type-I or Type-II MICA molecules.

**Results:**

Our analysis identified two major types of *MICA* polymorphisms based on antigenic epitopes and NKG2D binding characteristics. Type-I MICA characterized by six specific polymorphic site and their associated amino acid variants. exhibited significantly stronger NKG2D receptor binding affinity and more robust receptor activation compared to Type-II polymorphisms. This functional distinction was further corroborated by enhanced NK cells cytotoxicity against target cells expressing Type-I MICA molecules. Importantly, these differences in receptor activation and NK cell killing efficiency were attributable to six critical polymorphic amino acid sites.

**Conclusion:**

This study demonstrates the existence of two distinct types of *MICA* polymorphisms that differentially regulate NKG2D receptor activation and NK cell cytotoxicity. These findings offer new insights into that how genetic variation in MICA may contribute to individual differences in disease susceptibility through immune regulation mechanisms.

## 1 Introduction

The major histocompatibility complex class I-related gene A (*MICA*) is located on the short arm of human chromosome 6 within the major histocompatibility complex (MHC) class I gene region, adjacent to the *HLA-B* locus (1). This highly polymorphic gene exhibits its genetic variation primarily in the α1, α2, and α3 immunoglobulin-like extracellular structural domains, which are encoded by exons 2-4 (2). Current data from the IMGT/HLA database (https://www.ebi.ac.uk/ipd/imgt/hla/; version 3.57, 07-2024) document 576 human *MICA* alleles encoding 280 distinct protein variants. Under normal physiological conditions, MICA molecules exhibits low expression levels on the membranes of human epithelial cells (3), fibroblasts (4), and endothelial cells (5), with minimal to undetectable expression in other cell types. However, the expression of MICA is significantly upregulated in response to tumor transformation or viral infection (6, 7).

The main biological function of MICA is to serve as a ligand for the NKG2D (natural killer group 2 member D) receptor on the surface of natural killer (NK) cells, thereby activating NKG2D-mediated signaling pathway (8). Notably, amino acid polymorphisms in MICA have implicated in the pathogenesis of various diseases. For example, the single nucleotide polymorphism (SNP) rs2596542 is associated with an increased risk of hepatocellular carcinoma in hepatitis C virus (HCV) patients (9, 10) In patients with acute leukemia receiving hematopoietic stem cell transplantation, glycine (G) at position 14 of the MICA protein correlates with significantly decreased overall survival (11). Furthermore, a methionine-to-valine substitution at position 129 (M/V) substantially diminishes binding affinity to the NKG2D receptor (12), and this genotype significantly increases susceptibility to renal transplant rejection (13).

NK cell surface receptors consist of both activating and inhibitory types. Among these, the activating receptor NKG2D plays a pivotal role in immune surveillance: upon ligand engagement, it triggers NK cell activation, enhances cytokine secretion, and mediates cytotoxicity against target cells (14). The ligands for NKG2D, referred to as natural killer group 2 member D ligands (NKG2DL), include MICA, MICB (major histocompatibility complex class I chain-related protein B), and the UL-16 binding protein (ULBP) family. These ligands activate NK cells by binding to the NKG2D receptor on their surface (15). MICA exhibits remarkable polymorphism, with MICA variants displaying distinct binding affinities for NKG2D receptor (16). This polymorphism could modulate the activation potential of the NKG2D receptor, thereby regulating the cytotoxic activity of NK cells (17).

The NKG2D-NKG2DL pathway plays a critical role in immune regulation, with growing recognition of its therapeutic potential in cancer (18). Among NKG2DLs, MICA stands out as the most polymorphic member, exhibiting different binding affinities for the NKG2D receptor, which may result in divergent immune responses. This variability positions MICA as both a key immunoregulatory target and a significant contributor to autoimmune pathogenesis (15). Furthermore, as members of the MHC gene family, *MICA* alleles have been implicated in immune responses against allografts in organ transplant recipients, where the development of specific antibodies contributes to graft rejection (11). These findings suggest that MICA may represent an important target for immunoregulation strategies. However, the precise effects of MICA polymorphisms on the NKG2D receptor signaling pathway remains incompletely understood.

In this study, we identified 29 of the most common and representative MICA polymorphic molecules in the population which had two different response patterns. Multifactorial analysis of these response patterns and the amino acid sequence alignment of revealed the existence of two major types of MICA polymorphic molecules in the population, distinguished by six linked polymorphic amino acid sites and their corresponding residue types. Our findings provide mechanistic insights into how structural variations in MICA influence the activation ability of the NKG2D receptor, offering a new perspective for predicting susceptibility to individual disease.

## 2 Methods

### 2.1 Cell culture

NKG2D receptor reporter cells (NKG2D-2B4) were generated by our research team in collaboration with Prof. Chengcheng Zhang’s team at The University of Texas Southwestern Medical Center and are maintained in our laboratory (19). Hmy2.CIR cells were purchased from ATCC (USA), and MICA^+^Hmy2.CIR overexpressing cells were constructed by our team and are also maintained in our laboratory (19, 20). The NKL cell line was generously provided by M. J. Robertson’s team at the Indiana University School of Medicine, Indianapolis, IN (19). HEK 293F cells were purchased from Sino Biological.

All cell lines were cultured in RPMI 1640 medium (#11875500BT, Gibco, USA) supplemented with 10% fetal bovine serum (FBS, #10099141, Gibco, USA) and 1% penicillin/streptomycin (P/S, #P1400, Solarbio, China). NKL cells were additionally supplemented with 10 ng/mL interleukin-2 (IL-2, #200-02, PeproTech, USA). HEK 293F cells were cultured in serum-free 293-TII medium (#M293TII, Sino Biological, China) with shaking at 170 rpm. All cells were maintained at 37°C in a 5% CO_2_ incubator.

### 2.2 Soluble recombinant proteins

A total of 12 recombinant proteins, including MICA*045 (coated on Luminex beads), MICA*001, MICA*002, MICA*007, MICA*012, MICA*017, MICA*018, MICA*004, MICA*006, MICA*008, MICA*009, and MICA*019, were constructed in mammalian expression vectors in our laboratory. These proteins were then transfected into 293F cells, expressed, purified, and collected, respectively. Specifically, the signal peptide, extracellular protein sequence, and 6 × His tag sequences of the above 12 MICA proteins were ligated into the pcDNA 3.1(+) eukaryotic expression vector by double digestion Hind III (Cat#: R3104V, NEB, USA) and BamH I (Cat#: R3136V, NEB, USA). The expressing plasmid was transformed into Trans5α chemically competent cells (#CD201-01, Transgen, China), the recombinant cell colony was amplified, and the plasmids were extracted for sequencing verification. The plasmid was mixed with polyethyleneimine (25 kDa linear PEI, #23966, Polysciences, USA) at a 1:3 ratio (molecular weight) and transferred into 293F cells. After 3 days of expression, cell supernatants were collected, and the proteins were purified using affinity chromatography on nickel columns (Bestchrom, China).

About the NKG2D-Ig soluble recombinant protein, the extracellular nucleotide sequence of NKG2D and the human IgG Fc fragment were synthesized by the Beijing Genomics Institution. And the NKG2D-Ig fragment was ligated into the eukaryotic expression vector pFlag-CMV5.1 using T4 ligase (Cat#: EL0011, Invitrogen, USA) by double digestion. After transfecting the ligase product into Trans5α chemically competent cells, the recombinant cell colony was amplified, and the plasmids were extracted for sequencing verification. The protein was expressed and purified as above mentioned.

### 2.3 Monoclonal antibodies

The 11 recombinant MICA proteins (20 µg each) were mixed to form solutions of equal concentration and volume, which were then emulsified with Freund’s adjuvant as an antigen for immunizing BALB/c mice. Mice were immunized once a week for four consecutive weeks, after which they were sacrificed. Spleen cells were isolated from the mice and fused with mouse myeloma cells (SP2/0) to generate hybridoma cells. Monoclonal hybridomas were selected by limited dilution, expanded in culture, and the monoclonal antibodies were purified and collected. The specific methodology for producing monoclonal antibodies is described in the Zou et al. paper (21). One monoclonal antibody, named 5.2G1, was identified based on its dual response patterns to the MICA polymorphic molecules. The monoclonal antibody 6B3, which was kindly provided by the Southwestern Medical Center, USA, is maintained in our laboratory (21).

### 2.4 Serum samples

Human serum samples, S001 and S002, were obtained by our research team from the 16th International HLA and Immunogenetics Workshop (IHIW) and are stored in our laboratory (22). These serum samples were extracted from the peripheral blood of renal transplant recipients who had experienced postoperative humoral rejection.

### 2.5 MICA luminex array

Luminex beads containing 28 types of MICA polymorphic molecules were coated into Luminex Beads separately and assembled with a commission kits (Immunocore, USA). MICA*045 soluble protein was coated onto the beads following the manufacturer’s instructions, resulting in Luminex beads containing 29 different MICA polymorphic molecules. The MICA Luminex beads were diluted to 1 × 10^5^ beads/mL in 10% BSA solution, and 50 µL of this mixture was added to each well of a 96-well plate, with three replicate wells per group. Subsequently, 1 µg/mL of S001 serum, S002 serum, 5.2G1 monoclonal antibody (mAb), 6B3 mAb, mouse IgG, NKG2D-Ig fusion protein, and 10% BSA were added to the wells. The plate was incubated at room temperature for 40 minutes on a shaker set to 850 rpm, followed by centrifugation at 1500 rpm and washing with 100 µL of washing buffer. This washing step was repeated three times. Then, 50 µL of PE-conjugated goat anti-human/mouse IgG (#115-115-164, Jackson ImmunoResearch, USA) was added, and the plate was incubated for 30 minutes at room temperature on the shaker at 850 rpm. The beads were washed three times, resuspended in 50 µL PBS, and analyzed using a Luminex 200 instrument (Bio-Rad, USA).

### 2.6 SDS-PAGE, gel staining, and western blot

The NKG2D-Ig fusion protein was mixed with 6× loading buffer containing β-Mercaptoethanol and incubated in a water bath at 100°C for 5 minutes. A 20 µL sample was then loaded, along with a protein marker (#26619, ThermoFisher, USA), and separated by electrophoresis on a 10% SDS-PAGE gel at 100 V for 80 minutes.

For gel staining, the gel was stained with a 50% Coomassie Brilliant Blue solution (#R-250, Sigma, USA) for 30 minutes at room temperature, followed by destaining in Coomassie Brilliant Blue decolorizing solution, with shaking at 70 rpm at room temperature for 24 hours. The gel was then recorded.

For the western blot, the other piece of gel was transferred to a PVDF membrane. After electrophoretic transfer at 200 mA for 90 minutes, the membrane was blocked with 5% skimmed milk at 37°C for 1 hour. The membrane was subsequently incubated with 1:5000 HRP-conjugated goat anti-human IgG (#109-035-008, Jackson ImmunoResearch, USA) at 37°C for 30 minutes. After three washes with 0.05% PBST, chemiluminescent imaging was performed.

### 2.7 Amino acid sequence analysis of MICA polymorphic molecules

The amino acid sequences of 280 MICA polymorphic molecules from the IPD-IMGT/HLA database (https://www.ebi.ac.uk/ipd/imgt/hla/) (version 3.57, released July 2024) were analyzed using comparative clustering, with the MICA*001 sequence serving as the reference.

### 2.8 Flow cytometry

The cells were adjusted to a concentration of 1 × 10^6^ cells/mL in 2% FBS-PBS buffer, and the corresponding antibody was added at a 1:100 dilution. The cells were incubated for 30 minutes at 4°C, protected from light. After incubation, the cells were washed three times with buffer and then analyzed using a flow cytometer. For secondary antibody staining, the secondary antibody was added at a 1:500 dilution, incubated for 30 minutes at 4°C, protected from light, and washed three times with buffer before flow cytometry analysis.

To detect NKG2D molecules on NKG2D-2B4 and 2B4-mock cells, PE-conjugated anti-NKG2D antibody (#320806, Biolegend, USA) was used. Detection of NKG2D on NKL cells was performed with PE-conjugated anti-NKG2D antibody (#320806, Biolegend, USA), PE-conjugated Mouse IgG1, κ (#400111, Biolegend, USA), and PE-conjugated anti-mouse H-2 antibody (#125505, Biolegend, USA). For detecting MICA molecules on MICA^+^Hmy2.CIR and Hmy2.CIR cells, anti-MICA antibody (6B3) was used, followed by PE-conjugated anti-mouse IgG (H+L) antibody (#1034-09, SouthernBiotech, USA) and PE-conjugated anti-mouse H-2 antibody (#125505, Biolegend, USA).

### 2.9 NKG2D receptor reporter cell signaling pathway activation flow cytometry assay

The MICA recombinant protein solution was diluted to 80 µg/mL, and 50 µL of the solution was added to each well of a 96-well plate. The plate was incubated for 4 hours at 37°C, followed by two washes with PBS. NKG2D-2B4 and 2B4 cells were then added to the wells at a density of 8 × 10^4^ cells/well, with three replicate wells per group. In the soluble group, NKG2D-2B4 and 2B4 cells were seeded into uncoated 96-well plates at 8 × 10^4^ cells/well, and 50 µL of the MICA recombinant protein solution was added. The cells were cultured in a 37°C, 5% CO_2_ incubator for 16 hours. After incubation, the cells were collected by centrifugation, resuspended in 5% FBS-PBS buffer, and the proportion of GFP-positive cells was determined by flow cytometry. A dose-response curve was constructed with the dose on the horizontal axis and the percentage of GFP-positive reporter cells on the vertical axis.

### 2.10 NKG2D receptor-activated confocal immunofluorescence

50 µL of the MICA recombinant protein solution was added to each well of a 96-well plate and incubated at 37°C for 4 hours, followed by two washes with PBS. NKG2D-2B4 and 2B4 cells were seeded at a density of 8 × 10^4^ cells per well. The cells were pre-stained with anti-human NKG2D monoclonal antibody (eBioscience, USA) and incubated at 4°C for 30 minutes. After incubation, the cells were washed three times with PBS and then incubated with goat anti-mouse Fluor 594 (Invitrogen, USA) at 4°C for 30 minutes. In parallel, for the soluble group, 50 µL of the MICA recombinant protein solution was added to the uncoated wells, and the plate was incubated in a 5% CO_2_ incubator at 37°C for 16 hours. Finally, cells were observed under a confocal fluorescence microscope (Zeiss LSM 710).

### 2.11 NKL cell blocking and killing assay

MICA^+^Hmy2.CIR and Hmy2.CIR cells were stained with CFSE (CellTrace CFSE Cell Proliferation Kit, #C34554, ThermoFisher, USA) and then seeded into 96-well plates at a density of 5 × 10^4^ cells per well, followed by the addition of recombinant MICA soluble protein and NKG2D-Ig soluble protein at concentrations of 10 µg/mL for blocking and killing assays. NKL cells were added at E:T ratios of 1:1, 3:1, 5:1, and 10:1. After incubation at 37°C for 4 hours, the cells were stained with 7-AAD (#51-68981E, BD Pharmingen, USA) at a 1:500 dilution and analyzed by flow cytometry. Killing efficiency was calculated as the ratio of CFSE^+^ 7-AAD^+^ target cells, with CFSE-stained target cells serving as the 100% reference.

### 2.12 NKG2D allele sequencing

We collected 89 Chinese south Han healthy individuals to detected NKG2D allele frequency. 89 healthy individuals recruited from the Health Management Center of Xiangya Hospital, Central South University. All participants provided signed informed consent forms, and the study protocol was approved by the Ethics Committee of Xiangya Hospital, Central South University (approval number 201611608). Peripheral blood mononuclear cells (PBMCs) were isolated from 178 healthy donor blood samples using Ficoll (stemcell, Canada) density gradient centrifugation. Total RNA was extracted from PBMCs with a RNA extraction kit (Qiagen, China) following the manufacturer’s protocol. First-strand cDNA synthesis was performed using 1 μg total RNA with a reverse transcription kit (Biosharp, China) under recommended conditions: 37°C for 15 min followed by 85°C for 5 sec for enzyme inactivation.

NKG2D gene cDNA was amplified using 100 ng cDNA template with specific primers via PCR. The resulting products were purified and quantified to 20 ng/μL using nuclease-free DEPC-treated water. Sequencing libraries were prepared by mixing 40 μL DEPC water, 252 μL PCR Master Mix, and 8 μL high-fidelity Taq polymerase, followed by incubation at room temperature for 30 min. Amplification was performed under the following thermal procedure: 96°C, 2 min, then 96°C, 30 s, 20 cycles, 69°C for 50 s, 72°C for 90 s, then 72°C for 10 min to extension.

PCR products were electrophoresed (130 V, 30 min) in 1% agarose gel. Sequencing reactions utilized BigDye Terminator v3.1 Cycle Sequencing Kit with 4 μL template DNA and 2 μL primer per well, processed through: 25 cycles: 96°C for 10 s → 50°C for 5 s → 60°C for 2 min.

Post-sequencing cleanup involved SDS treatment (1.5 μL 2% SDS/well) and Sephadex G-50 column purification. Samples were analyzed on an ABI 3730xl Genetic Analyzer, with sequences aligned to reference NKG2D alleles (GenBank: NM_001349433.1) using CodonCode Aligner v10.0.

### 2.13 Statistical analysis

The experimental data were statistically analyzed using SPSS 22.0 software. Measurement data are presented as mean ± SD, and comparisons between two groups were made using an independent sample *t*-test (non-parametric and unpaired tests). The allele frequencies of *MICA* were estimated based on the principles of the Poisson distribution. Graphs were generated using GraphPad Prism 9.0. A *P*-value of < 0.05 was considered statistically significant. *P < 0.05, **P < 0.01, ***P < 0.001.

## 3 Result

### 3.1 More than 90% of the population carries one or two of the 29 common *MICA* polymorphic alleles

A total of 280 MICA polymorphic molecules have been identified in the population. Since the distribution of *MICA* alleles is not evenly distributed across the population, the numbers of the common *MICA* alleles (frequency > 1%) are limited among the populations. In this study, we selected a Luminex bead array kit containing 28 MICA polymorphic proteins, along with the MICA*045 allele protein, which is prevalent in the Chinese population. These 29 proteins were conjugated into a Luminex beads respectively as MICA antigens array, forming a liquid microarray detection platform for testing. A search of the PubMed database was conducted for MICA allele frequency studies across various populations, and statistical analysis was performed. Based on the combined allele frequencies of the 29 MICA polymorphisms and the frequency of prevalent MICA*010 allele, which encodes a non-expressing protein, it was concluded that the proportion of populations carrying one or two of the 29 MICA polymorphic molecules ranged from 91.6% to 99.4% in different regional populations (**Table 1**). The 29 MICA polymorphic proteins selected can cover for more than 90% population (**Supplementary Material 1**).

**Table 1:** The Frequency of Three Groups of *MICA* Alleles in Different Populations.

*MICA* allele	Chinese (25) (N=144)	American (26) (N=103)	Japanese (27) (N=130)	European (28) (N=154)	African (29) (N=201)
The 29 *MICA^a^*	73.27	84.60	88.10	90.59	98.70
MICA*010^b^	22.22	7.00	10.80	4.55	0.70
other	4.51	8.40	1.20	4.86	0.60
Total	100	100	100	100	100

a. The combined frequency of 29 common *MICA* alleles. b. The MICA*010 allele is not expressed on the cell membrane but is common in population.

### 3.2 Presence of two broadly specific antigenic epitopes in the human polymorphic MICA molecule

**Figure 1:**
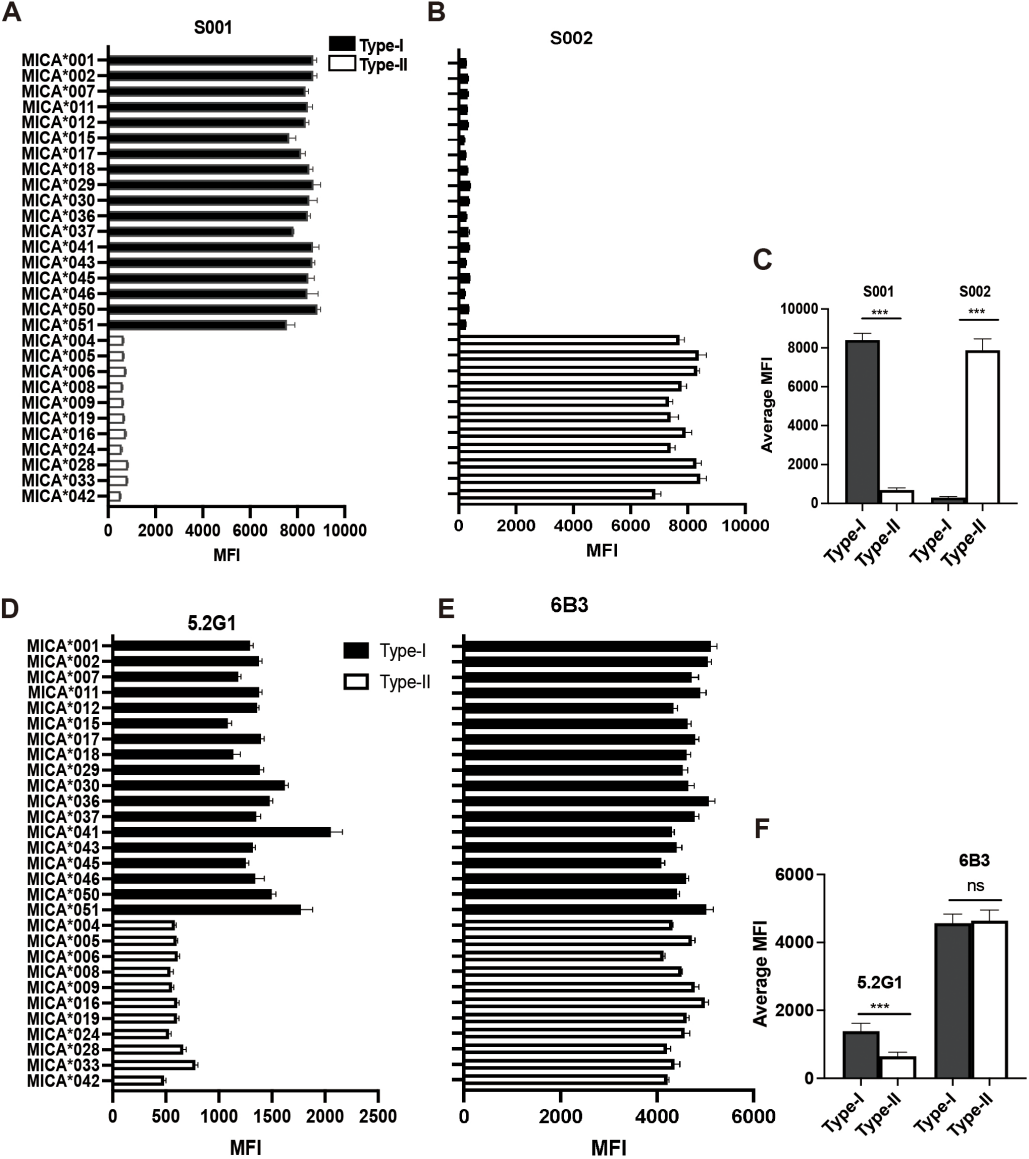
Human MICA polymorphic proteins exhibit two major opposing antigenic epitopes. **(A)** Reactivity characteristics of 29 MICA polymorphic molecules with S001 serum. **(B)** Reactivity characteristics of 29 MICA polymorphic molecules with S002 serum. **(C)** Comparison of the average binding ability of S001 and S002 serum to Type-I and Type II MICA proteins of 29 MICA polymorphic proteins, with statistical analysis performed using independent sample *t*-test. **(D)** Reactivity characteristics of 29 MICA polymorphic molecules with monoclonal antibody 5.2G1. **(E)** Reactivity characteristics of 29 MICA polymorphic molecules with monoclonal antibody 6B3. **(F)** Comparison of the average binding ability of 5.2G1 and 6B3 monoclonal antibody to Type-I and Type II MICA proteins of 29 MICA polymorphic proteins, with statistical analysis performed using independent sample *t*-test. ***P<0.001; ns, not significant.

The two serum samples (S001 and S002) obtained from the renal transplant recipients had been characterized to contain allo-antibodies with different specificities (23). Using a 29 MICA polymorphic molecules Luminex array, the results revealed that serum S001 showed a strong positive reaction with 18 MICA polymorphic proteins, including MICA*001, MICA*002, MICA*007, MICA*011, MICA*012, MICA*015, MICA*017, MICA*018, MICA*029, MICA*030, MICA*036, MICA*037, MICA*041, MICA*043, MICA*045, MICA*046, MICA*050, and MICA*051, with an average mean fluorescence intensity (MFI) value of 8380.48 ± 351.01. While S001 serum showed a low positive reaction with 11 other MICA polymorphic proteins, including MICA*004, MICA*005, MICA*006, MICA*008, MICA*009, MICA*016, MICA*019, MICA*024, MICA*028, MICA*033, and MICA*042, with an average MFI value of 680.64 ± 93.54 (P < 0.001) (**Figures 1A, C**, **Table 2**). Conversely, S002 serum exhibited a completely opposite reactivity pattern to S001 serum, showing high reactivity with the MICA polymorphic molecules that had lower MFI values in S001, and low reactivity with the group of MICA polymorphic molecules that had higher MFI values in S001. The MFI values tested by these two groups of MICA molecules with S002 were 307.89 ± 55.45 and 7806.39 ± 498.21, respectively (P < 0.001) (**Figures 1B, C**, **Table 2**). These results suggest that, despite the high polymorphism of MICA molecules within the population, the antigen-antibody reactions indicate the presence of two major reciprocal antigenic epitopes.

**Table 2:** MFI values of the two major types of MICA in response to anti-MICA antibodies and the NKG2D receptor.

Tested	Type I(N=18)	Type II(N=11)	*P*
	Mean ± SD	Mean ± SD	
human Ab S001	8380.48 ± 351.01	680.64 ± 93.54	<0.0001
human Ab S002	307.89 ± 55.45	7806.39 ± 498.21	<0.0001
mAb 5.2G1	1405.06 ± 223.11	597.55 ± 73.54	<0.0001
NKG2D-Ig	1977.52 ± 446.00	655.70 ± 175.68	<0.0001

In addition, during the identification of 11 mouse anti-human MICA monoclonal hybridoma cell strains, we observed that the monoclonal antibody produced by one of the hybridoma strains (designated 5.2G1) exhibited a reactive pattern against the 29 MICA polymorphic molecules that closely resembled the reactivity observed in the human allo-antibodies of serum S001. The MFI value for 5.2G1 with the two groups of MICA polymorphic molecules was 1405.06 ± 223.11 vs. 597.55 ± 73.54 (P < 0.001) (**Figures 1D, F**, **Table 2**). While the general monoclonal antibody 6B3 showed a strong, uniform positive response across all MICA polymorphic molecules in the Luminex beads array, yielding a consistent and broad reactivity pattern (**Figures 1E, F**). These results confirm the presence of broadly specific antigenic epitopes among human MICA polymorphic molecules.

**Figure 2:**
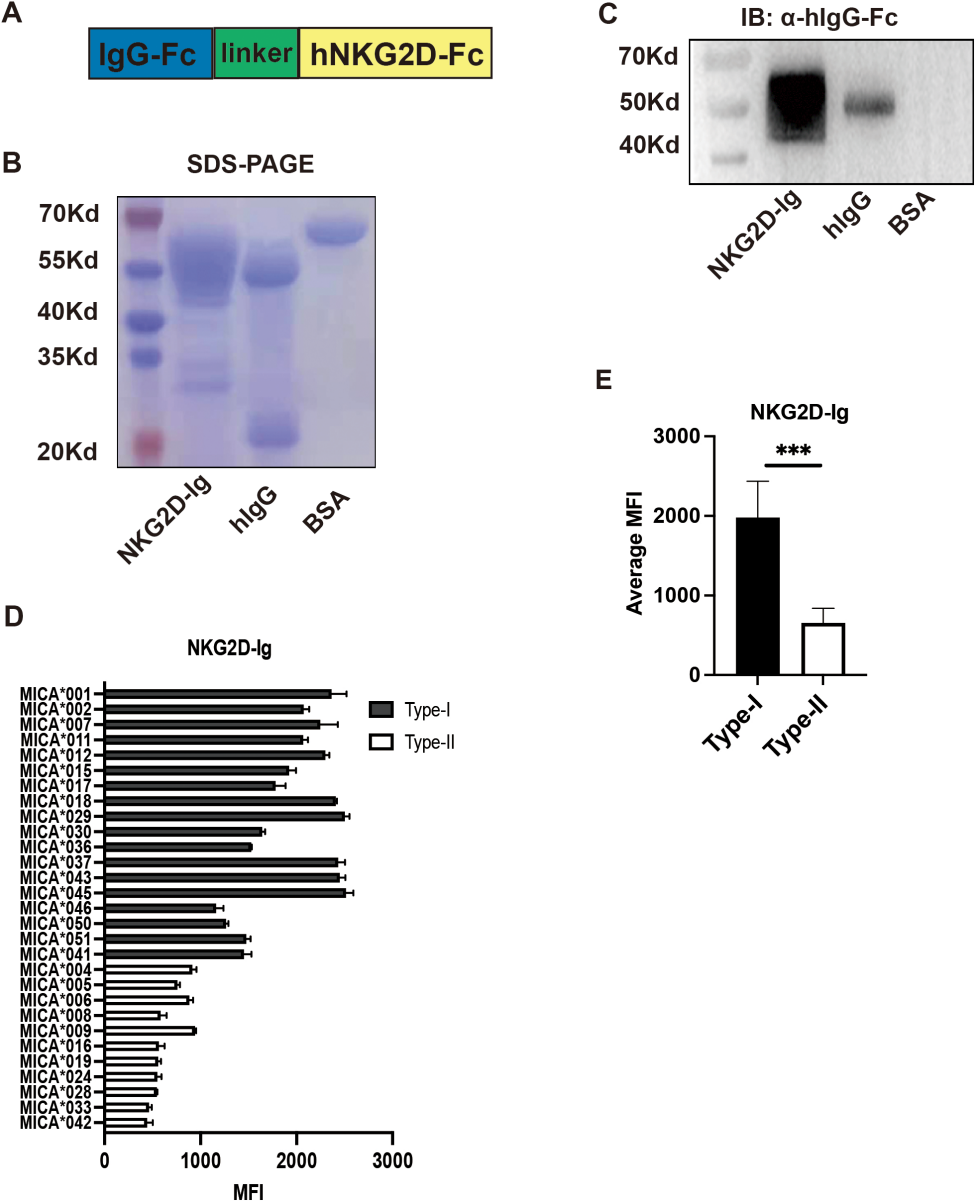
Preparation and Functional Detection of NKG2D-Ig Fusion Protein. **(A)** Schematic diagram of the structure of NKG2D-Ig fusion protein, with the N-terminal on the left and C-terminal on the right. **(B)** Coomassie brilliant blue staining showing the molecular size of the NKG2D-Ig fusion protein, with a molecular weight of 46 kDa, using human IgG and bovine serum albumin (BSA) as controls. **(C)** Detection of NKG2D-Ig fusion protein using anti-human IgG Fc antibody by Western blot, with a protein size of approximately 46 kDa, using human IgG and BSA as controls. **(D)** Reactivity characteristics of 29 MICA polymorphic molecules with NKG2D-Ig fusion protein. **(E)** Comparison of the binding ability of Type-I and Type-II proteins of 29 MICA polymorphic proteins, with statistical analysis performed using independent sample *t*-test.***P<0.001.

The primary biological function of MICA molecules is to activate the NKG2D receptor. Based on this, we hypothesized that the broad-specific antigenic epitopes present in MICA polymorphic molecules may exhibit differential binding affinities for the NKG2D receptor, thereby influencing NK cell activation. To test this hypothesis, we prepared soluble NKG2D receptor fusion proteins (NKG2D-Ig) (**Figure 2A**). The purified soluble NKG2D fusion proteins displayed a band at the molecular weight of 46 kDa with SDS-PAGE, and Coomassie brilliant blue staining. Meanwhile, the hIgG antibody displayed a band of heavy chain at the molecular weight of 55 kDa and a band of light chain at the molecular weight of 25 kDa. The BSA protein displayed a band at the molecular weight of 66 kDa (**Figure 2B**). Additionally, the soluble NKG2D receptor fusion protein with a flag of Fc fragment of human IgG was confirmed as the same molecular weight of 46 kDa (**Figure 2C**).

Subsequently, the NKG2D-Ig protein was incubated with the 29 MICA Luminex beads array, and the MFI value was used as an index to assess the binding affinity between the NKG2D receptor and the MICA polymorphic molecules. The results revealed two distinct reactivity patterns for the NKG2D-Ig protein against the two major groups of MICA polymorphic molecules (1977.52 ± 446.00 vs. 655.70 ± 175.68, P < 0.0001) (**Figures 2D, E**, **Table 2**). This reactivity pattern closely consistent with the reactivity observed with human serum S001 and monoclonal antibody 5.2G1, further confirming the different binding affinity among the two major groups of the 29 MICA polymorphic molecules.

### 3.3 Response pattern of NKG2D receptor against two major groups of MICA is dependent on key polymorphic sites and its amino acid types in MICA molecules

We analyzed the binding affinities of allo-antibodies and the NKG2D-Ig protein to the 29 MICA polymorphic molecules, suggesting the presence of two major types of specific epitopes. Then that was confirmed by mAb 5.2G1. To further investigate this, we compared the amino acid polymorphic sites and their corresponding amino acid variants in these two groups of MICA polymorphic molecules by using data from the HLA/IMGT database. There have 27 amino acid polymorphic sites that were located in the extracellular membrane region of these MICA molecules in the MICA polymorphic amino acid alignment (**Table 3**). The MICA polymorphic molecules with high binding affinity for the NKG2D receptor were strongly correlated with six polymorphic sites and linked with six amino acid types: C_36_+M_129_+K_173_+G_206_+W_210_+S_215_, we call it as Type I MICA. While the six linked amino acid species is Y_36_+V_129_+E_173_+S_206_+R_210_+T_215_, is classified as Type II MICA. Type II MICA polymorphic molecules exhibited a lower binding affinity for NKG2D than Type I MICA molecules (**Table 2**). Interestingly, human MICA polymorphic molecules are classified into either Type I or Type II. Other polymorphisms sites in the MICA molecules, and their corresponding amino acid variants, did not influence the classification of these two major MICA types.

**Table 3:** Amino acid sequence alignment of 29 MICA polymorphic molecules.

		polymorphic site																										
MICA-		0	0	0	0	0	0	1	1	1	1	1	1	1	1	1	1	1	1	2	2	2	2	2	2	2	2	2
		1	2	2	3	9	9	0	1	2	2	2	4	5	5	7	7	7	8	0	0	1	1	1	2	5	5	7
		4	4	6	6	0	1	5	4	2	5	9	2	1	6	3	5	6	1	6	8	0	3	5	1	1	6	1
	001	W	T	V	C	L	Q	R	G	L	K	M	V	M	H	K	G	V	T	G	Y	W	T	S	V	Q	R	P
Type-I	002	G	A	–	-	–	–	–	–	–	E	-	–	–	–	-	–	–	–	-	–	-	–	-	–	–	–	–
	007	–	A	–	-	–	–	–	–	–	E	-	–	–	–	-	–	–	–	-	–	-	–	-	–	–	–	–
	011	G	A	–	-	–	–	–	–	–	E	-	–	V	–	-	–	–	–	-	–	-	–	-	–	–	–	A
	012	–	–	–	-	–	–	–	–	–	E	-	–	–	L	-	–	–	–	-	–	-	–	-	–	–	–	–
	015	G	A	–	-	–	–	–	R	–	E	-	–	–	–	-	–	–	–	-	–	-	–	-	–	–	–	–
	017	G	A	–	-	–	R	–	–	–	E	-	–	–	–	-	–	–	–	-	–	-	–	-	–	–	–	–
	018	–	–	–	-	–	–	–	–	–	E	-	–	–	–	-	–	–	–	-	–	-	–	-	–	–	–	–
	029	–	A	–	-	–	–	–	–	–	E	-	I	–	–	-	–	–	–	-	–	-	–	-	–	–	–	–
	030	G	A	–	-	–	–	–	–	–	E	-	–	–	–	-	–	–	–	-	–	-	–	-	–	–	–	A
	036	G	A	–	-	–	–	K	–	–	E	-	–	–	–	E	S	–	–	-	–	-	–	-	–	–	–	–
	037	–	A	–	-	–	–	–	–	–	E	-	–	–	–	-	–	–	–	S	–	R	I	T	–	R	–	–
	041	G	A	G	-	–	–	–	–	–	E	-	–	–	–	-	–	–	–	-	–	-	–	-	–	–	–	–
	043	–	A	–	-	–	–	–	–	–	E	-	–	–	R	-	–	–	–	-	–	-	–	-	–	–	S	–
	045	–	A	–	-	–	–	–	–	–	E	-	–	–	–	-	–	–	–	-	–	-	–	-	–	E	–	–
	046	G	A	–	-	–	–	–	–	–	E	-	–	–	–	-	–	–	–	-	C	-	–	-	–	–	–	–
	050	G	A	–	-	F	–	–	–	–	E	-	–	–	–	-	–	–	–	-	–	-	–	-	–	–	–	–
	051	–	A	–	Y	–	–	–	–	–	E	-	–	–	–	-	–	–	–	-	–	-	–	-	–	–	–	–
Type-II	004	–	A	–	Y	–	–	–	–	V	E	V	–	–	–	E	S	–	R	S	–	R	–	T	–	–	–	–
	005	–	A	–	Y	–	–	–	–	–	E	V	–	–	–	-	–	–	–	S	–	R	–	T	–	R	–	–
	006	–	A	–	Y	–	–	–	–	V	E	V	–	–	–	E	S	I	–	S	–	R	–	T	–	–	–	–
	008	–	A	–	Y	–	–	–	–	–	E	V	–	–	–	E	–	–	–	S	–	R	I	T	–	R	–	–
	009	–	A	–	Y	–	–	–	–	V	E	V	–	–	–	E	S	–	–	S	–	R	–	T	–	–	–	–
	016	–	A	–	Y	–	–	–	–	–	E	V	–	–	–	E	–	–	–	S	–	R	–	T	L	–	–	–
	019	–	A	–	Y	–	–	–	–	–	E	V	–	–	–	E	S	–	–	S	–	R	I	T	–	R	–	–
	024	–	A	–	Y	–	–	–	–	–	E	V	–	–	–	E	–	–	–	S	–	R	–	T	–	–	–	–
	028	–	A	–	Y	–	–	–	–	–	E	V	–	–	–	E	–	–	–	-	–	-	–	-	–	–	–	–
	033	–	A	–	Y	–	–	–	–	–	E	V	–	–	–	E	S	–	–	S	–	R	I	T	–	R	–	–
	042	–	A	–	Y	–	–	–	–	–	E	-	–	–	–	-	–	–	–	S	–	R	I	T	–	R	–	–

Polymorphic sites of amino acid residues in MICA protein molecules, with non-polymorphic sites in the sequence not included. MICA*001 was used as the reference sequence. “-” indicates that the amino acid residue at this position is identical to that in MICA*001.

The orange color in the headline means the six-linked polymorphic sites. The blue color of amino acid type means Type-I MICA molecules. The green color of amino acid type means Type-II MICA molecules.

### 3.4 Type-I MICA polymorphic molecules activated the signaling pathway of the NKG2D receptor at a significantly lower dose than type-II MICA polymorphic molecules

**Figure 3:**
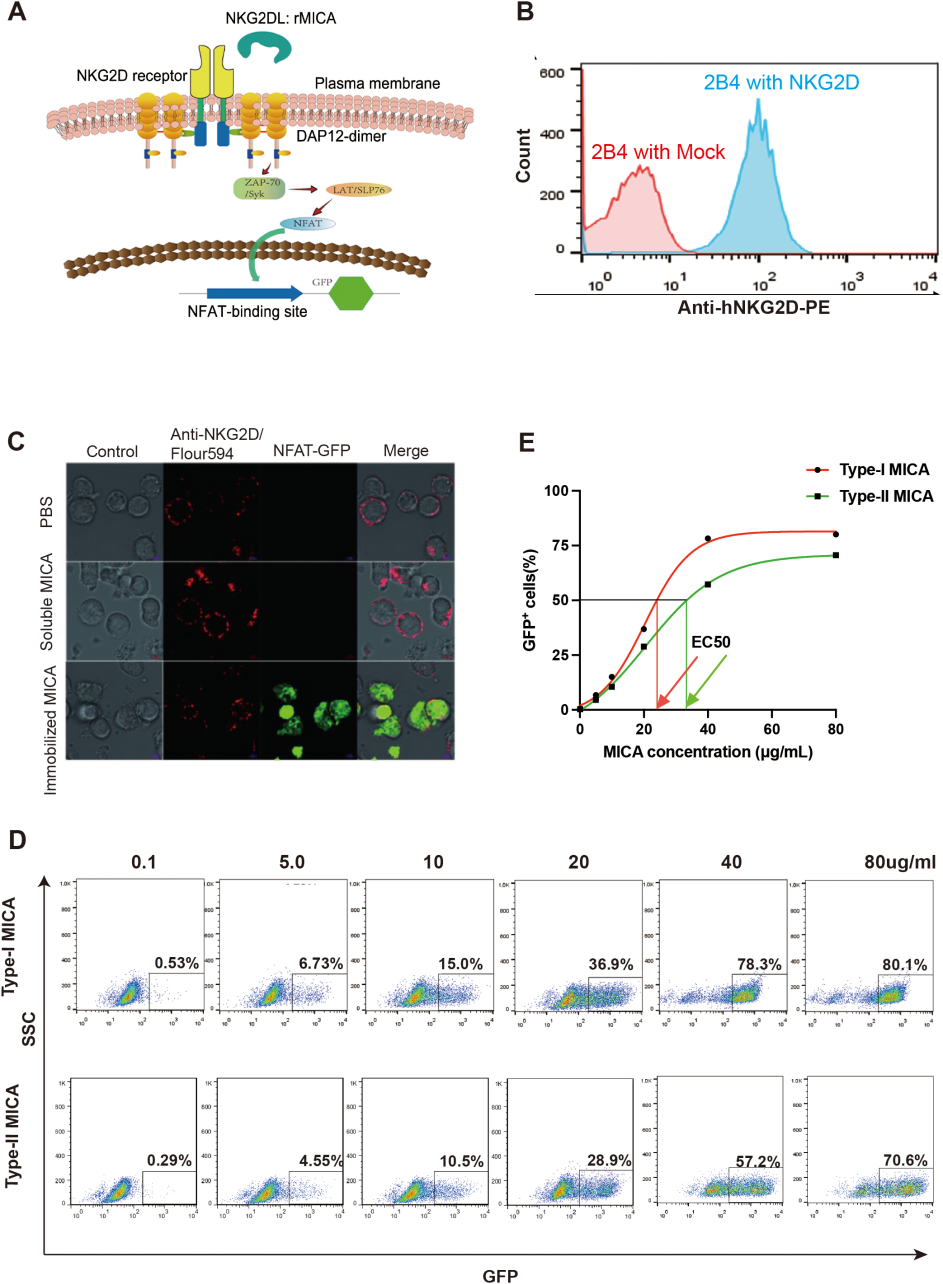
There are differences in the ability of the two major types of MICA polymorphic molecules to activate the NKG2D receptor. **(A)** Schematic diagram of the structure of NKG2D receptor reporter cell. **(B)** The NKG2D receptor reporter cells stably express the NKG2D molecule on the surface. **(C)** Immobilized MICA protein could activate NKG2D reporter cell to produce GFP. **(D)** The percentage of GFP^+^ reporter cells activated by different concentration of MICA polymorphic molecules. **(E)** The dose-response curves to evaluate the activation of the NKG2D receptor.

The above experiments confirmed the existence of differential affinities between the two major types of MICA polymorphic molecules binding with the NKG2D receptor. To test whether this variation in ligand-receptor binding may relate to NKG2D receptor activation, we utilized an NKG2D receptor reporter cell line (NKG2D-2B4), which was previously constructed in our laboratory. The NKG2D receptor reporter cells were constructed by using a human-mouse chimeric receptor structure, with the extracellular domain of human NKG2D receptor and the intracellular domains of the murine NKG2D receptor, enabling interaction with the DAP-12 adapter protein of the murine-derived 2B4 T hybridoma cells, which forward to activate the downstream signaling pathways. Upon binding of its ligand, the NKG2D receptor triggers the activation of downstream signaling pathways, resulting in the expression of GFP and the emission of green biofluorescence (**Figures 3A, B**). We selected the most common MICA polymorphic proteins in humans (MICA*002 belonging to Type I and MICA*008 to Type II) for dose-response experiments to evaluate the activation of the NKG2D signaling pathway. Our prior studies have shown that soluble MICA proteins can bind to the NKG2D receptor but do not activate the NKG2D signaling pathway (19). To address this, soluble MICA proteins were pre-coated onto a 96-well plate, followed by the addition of NKG2D receptor reporter cells to assess activation with biofluorescence (**Figure 3C**). The dose of immobilized MICA was positively correlated with the proportion of GFP^+^ cells, which was detected by flow cytometry (**Figure 3D**). Dose-response curves were generated to represent the proportion of activated reporter cells (GFP^+^ cells) induced by different concentrations of Type I and Type II proteins. The concentration of MICA protein at the point of 50% of NKG2D reporter cells (GFP^+^) reported biofluorescence was defined as the EC 50 value. The results indicated that the EC 50 value for Type I molecules was 24.97 μg/mL, while for Type II molecules, it was 37.34 μg/mL, which means that the lower the EC 50 value was, the stronger the activate ability of the MICA molecules was (**Figure 3E**).

Using the dose-response curves described earlier, we applied the 11 MICA polymorphic molecules to react with NKG2D receptor reporter cells at six concentrations and determined the EC 50 values for each, as derived from the curves. Among them, Type I MICA molecules (n=6) include MICA*001, MICA*002, MICA*007, MICA*012, MICA*017, and MICA*018, with an average EC 50 value of 24.95 ± 3.14 μg/mL. Type II MICA molecules (n=5) include MICA*004, MICA*006, MICA*008, MICA*009, and MICA*019, with a mean EC 50 value of 39.68 ± 4.46 μg/mL. The EC 50 value for Type I MICA molecules was significantly lower than that of Type II MICA molecules (P < 0.001, **Table 4**), indicating that a lower dose (24.95 μg/mL) of Type I MICA polymorphic molecules was sufficient to activate 50% of the NKG2D receptor reporter cells (50% GFP^+^), while a higher dose (39.68 μg/mL) of Type II MICA polymorphic molecules was required to achieve the same level of activation (50% GFP^+^).

**Table 4:** The dose of 11 MICA polymorphic molecules that activates 50% of the NKG2D receptor response (EC50).

MICA polymorphic molecules		Average EC 50 (μg/mL)	$\bar{\text{X}}$ (μg/mL)	*P* value
Type-I (N=6)	001	26.35	24.95	<0.001
	002	21.96		
	007	25.16		
	012	28.61		
	017	21.55		
	018	26.04		
Type-II (N=5)	004	39.54	39.68	
	006	38.67		
	008	41.84		
	009	34.84		
	019	43.49		

### 3.5 The killing efficiency of NKL cells against MICA stably expressing cells with type-I MICA was significantly higher than that with type-II MICA

**Figure 4:**
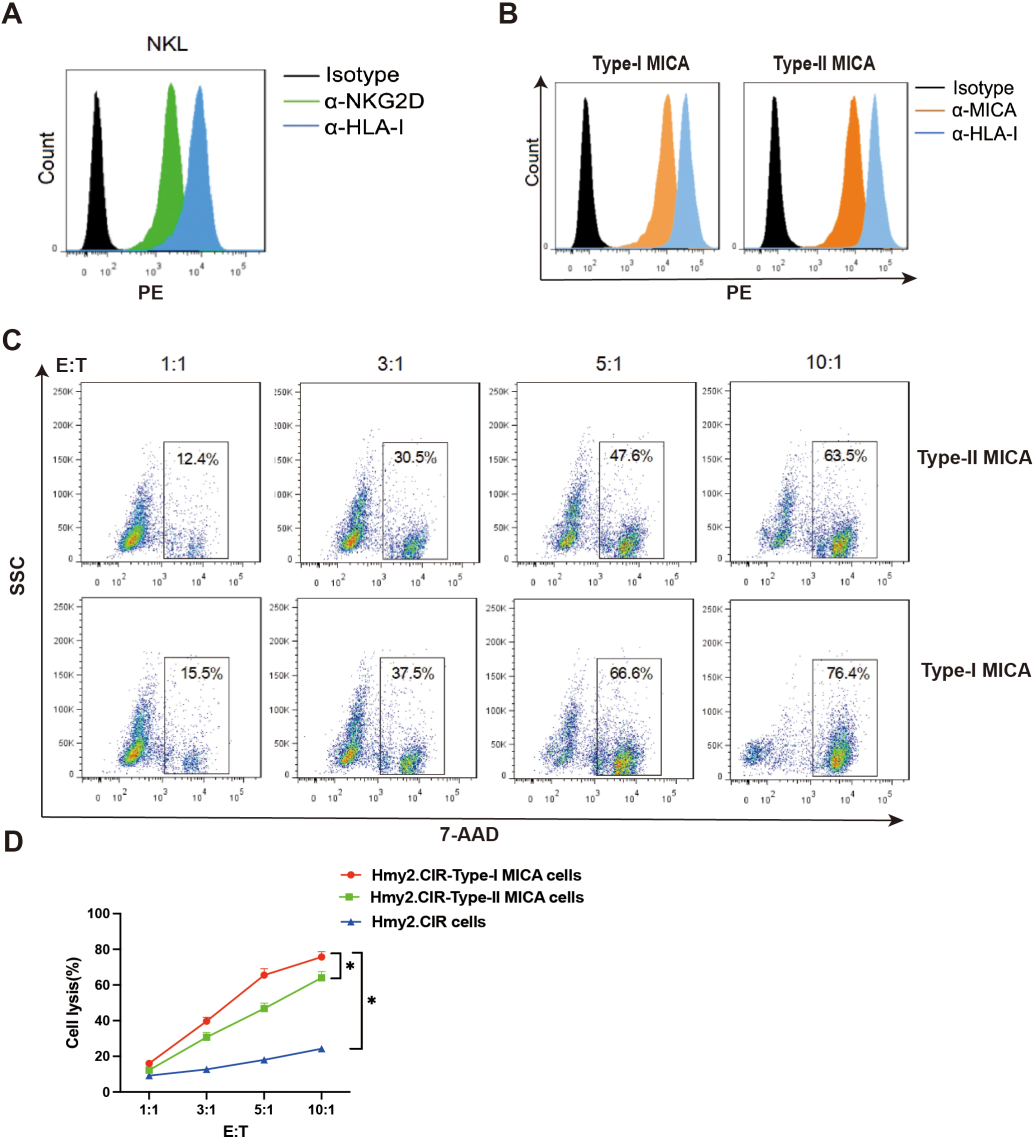
The killing efficiency of NKL cells against Type-I MICA cells is higher than that against Type-II MICA cells. **(A)** The NKL cells stably express the NKG2D molecule on the surface. **(B)** The two types of MICA overexpressing Hmy2.CIR cells stably and evenly express the MICA molecule on the surface. **(C, D)** The killing efficiency of NKL cells against the two types of MICA^+^ Hmy2.CIR cells. *P<0.05.

The NKG2D receptor on the NKL cell membrane was detected using an anti-NKG2D monoclonal antibody, demonstrating high expression of the NKG2D receptor (**Figure 4A**). Our previous studies have shown that NKL cells function as effector cells with a specific killing effect on human B lymphoblastoid cells that overexpress MICA. This cytotoxic effect can be inhibited by NKG2D-Ig fusion proteins or soluble recombinant MICA proteins, resulting in a significant reduction in killing efficiency (20). We also genotyped the *NKG2D* gene in a healthy population (**Supplementary Material 2**) and identified four distinct alleles; however, no amino acid sequence changes were found, indicating that the NKG2D receptor protein is conserved across human individuals. Thus, it can be inferred that the NKG2D receptor expressed on the NKL cell line serves as a reliable model for assessing the killing efficiency of MICA-expressing target cells in the populations.

To evaluate the cytotoxic efficiency of NKL cells, we constructed human B-lymphoblastoid cells (Hmy2.CIR) overexpressing Type-I MICA (MICA*002) and Type-II MICA (MICA*008) molecules. These overexpressing cells were stained with an anti-MICA monoclonal antibody, and two cell strains with comparable membrane expression levels of Type-I and Type-II MICA molecules were selected and expanded as target cells (**Figure 4B**). Flow cytometry was then used to assess the killing efficiency of NKL cells against these target cells. The proportion of killed target cells (7-AAD^+^) was calculated at different effector-to-target (E: T) ratios (1:1, 3:1, 5:1, and 10:1), and killing curves were plotted for NKL cells targeting cells stably expressing either Type-I or Type-II MICA. The results showed that NKL cells exhibited a significantly higher killing efficiency against Type-I MICA target cells compared to Type-II MICA target cells (P < 0.05, **Figures 4C, D**).

## 4 Discussion

To investigate the functional differences among various MICA polymorphic molecules, we selected 29 representative variants based on their reactivity to anti-MICA antibodies and NKG2D receptor. These polymorphisms correspond to alleles found in more than 90% of the population. Although MICA*010 is present in the population, it was excluded from this study due to its lack of protein expression (24). The 29 MICA polymorphic molecules selected for structural and functional analysis in this study are considered broadly representative of the population’s diversity (25–29).

Variations in the activation of NKG2D receptor by MICA polymorphic molecules may be attributed to specific amino acid polymorphisms. In our previous study, we identified two opposing antigen-antibody reaction profiles in the serum samples from renal transplant patients obtained from ASHI (23). Building on this, our current study further confirmed that the 29 MICA polymorphic molecules exhibited two opposing antigen-antibody response profiles when exposed to allo-antibodies from two renal transplant recipients. Similar dichotomous patterns were also observed in reaction with anti-MICA monoclonal antibodies. These findings suggest the existence of two major types of MICA polymorphic molecules. Notably, these two MICA phenotypes displayed distinct binding affinities to soluble NKG2D receptor proteins.

Most previous studies primarily focused on single amino acid polymorphisms in MICA molecules, particularly the well-characterized M/V dimorphism at position 129. This variant has been linked to differential binding affinity to the NKG2D receptor, with the M variant conferring significantly higher affinity compared to the V variant (13, 30). However, subsequent studies have indicated that the NKG2D binding affinity is not solely determined by the MICA-129 allele (23, 31). In our study, cluster analysis of the amino acid polymorphisms across all 280 MICA variants revealed two major groups characterized by six linked polymorphic residues: position 36, 129, 173, 206, 210, and 215. Notably, the residue at position 129 was found to be tightly linked to these sites. When position 129 is M, the six linked amino acids at the six sites typically form the sequence C_36_+M_129_+K_173_+G_206_+W_210_+S_215_. MICA polymorphic molecules with this combination exhibit higher binding affinity to both antibodies and the NKG2D receptor. In contrast, when position 129 is V, the linked amino acids at these six sites are usually Y_36_+V_129_+E_173_+S_206_+R_210_+T_215_, resulting in a lower binding affinity to the NKG2D receptor. Consequently, MICA polymorphic molecules with high binding affinity and these six linked amino acids were classified as Type-I MICA, while those with lower affinity were classified as Type-II MICA. The extracellular domains of MICA consist of the α1, α2, and α3 immunoglobulin-like structural domains, with the α1 and α2 domains being primary responsible for NKG2D receptor binding. The amino acids at positions 36, 129, and 173, located within the α1 and α2 domains, are key components of antigenic epitopes, forming the molecular structural basis for distinguishing the two major types of MICA (31, 32).

To investigate whether there is a significant difference in the ability of the two of MICA polymorphic phenotypes to activate the NKG2D receptor signaling pathway, we stimulated NKG2D receptor reporter cells with recombinant MICA proteins at varying concentrations. The EC50 value, defined as the concentration at which 50% of the reporter cells exhibited biofluorescence (GFP^+^), was determined.

As a key effector cell type in innate immunity, NK cells play a crucial role in the early-stage elimination of tumor cells and virus-infected cells. The NKG2D receptor is essential for NK cell activation and transduces signals through the DAP10 adaptor via multiple signaling pathways. NKG2D can initiate various forms of signal transduction via phosphorylation, activating mitogen-activated protein kinase (MAPK) and Janus kinase (Jak)/signal transducer and activator of transcription (STAT) signaling pathways (33). NKG2D ligands, such as MICA, regulate the receptor function, with upregulation of MICA expression leading to increased NKG2D receptor expression (34). The variable affinity of NKG2D for MICA may influence receptor activation in predisposed individuals, which has been observed in various autoimmune diseases (34). The affinity of the NKG2D receptor for MICA is higher than that for other ligands. However, mutations in certain MICA sites can reduce the formation of hydrogen bonds, thereby suppressing NKG2D receptor-mediated NK cell activation (35). In this study, we found that Type-I and Type-II MICA molecules differed in their ability to activate the NKG2D receptor signaling pathway. These findings indicate that the two major MICA polymorphic types not only vary in their binding affinity to the NKG2D receptor but also in their capacity to activate the NKG2D signaling pathway. Furthermore, our results demonstrate that these two MICA phenotypes also differ in the efficiency of NKL cell-mediated cytotoxicity, highlighting distinct molecular mechanisms that regulate NK cell activation and target cell killing.

The NKG2D receptor is encoded by the highly conserved *KLRK1* gene, which exhibits limited polymorphisms (36). While certain variants have been implicated in diseases such as rheumatoid arthritis (RA) and HPV-induced cancers, their causal relationships and underlying mechanisms remain unclear (37, 38). Our SBT sequencing analysis of *KLRK1* transcripts across study populations detected no amino acid-altering polymorphisms suggesting that the structural variation in NKG2D receptor itself does not account for observed differences in receptor activation. Rather these functional variations appear to stem primarily from polymorphisms in the MICA molecules. Although, the NKG2D receptor interacts with multiple ligands-including the MICB and ULBP families- MICA is considered the most important due to its highest degree of polymorphism and its dominant role in receptor activation (15). The differential effects of the NKG2D-MICA axis in innate immune responses are primarily determined by MICA polymorphisms.

In summary, our results are the first to identify two major types of MICA polymorphic molecules distinguished by six linked amino acid sites, offering insight into how MICA polymorphism regulates the NKG2D signaling pathway. Based on these findings, individuals can be classified into three MICA phenotypic groups: Type-I homozygote, Type-II homozygote, and Type-I/Type-II heterozygote. Since Type-I MICA ligands more efficiently activate the NKG2D receptor on NK cells compared to Type-II ligands, individuals with the Type-I homozygous phenotype may exhibit heightened NK cell responsiveness. This could enhance protection against infections and tumors but may also increase susceptibility to autoimmune diseases (39). Conversely, individuals with the Type-II homozygous phenotype may show reduced NK cell activation and potentially exhibit the opposite disease susceptibility. The relationship between *MICA* phenotypes and disease outcomes across these three groups remains unclear, and further clinical data are necessary to explore these correlations. Our findings provide valuable insights into the molecular and functional regulation of the NKG2D-MICA axis, which demand further in-depth investigation.

## Data Availability

The original contributions presented in the study are publicly available. This data can be found here: https://www.ncbi.nlm.nih.gov/sra/PRJNA1260497.
