# Supplementary figures and images for: Two major human phenotypes of MICA molecules and their differential activation to NK cells via NKG2D receptor
Source: Front Immunol. 2025 May 19;16:1563872. doi: 10.3389/fimmu.2025.1563872 (PMC12127157; doi:10.3389/fimmu.2025.1563872)

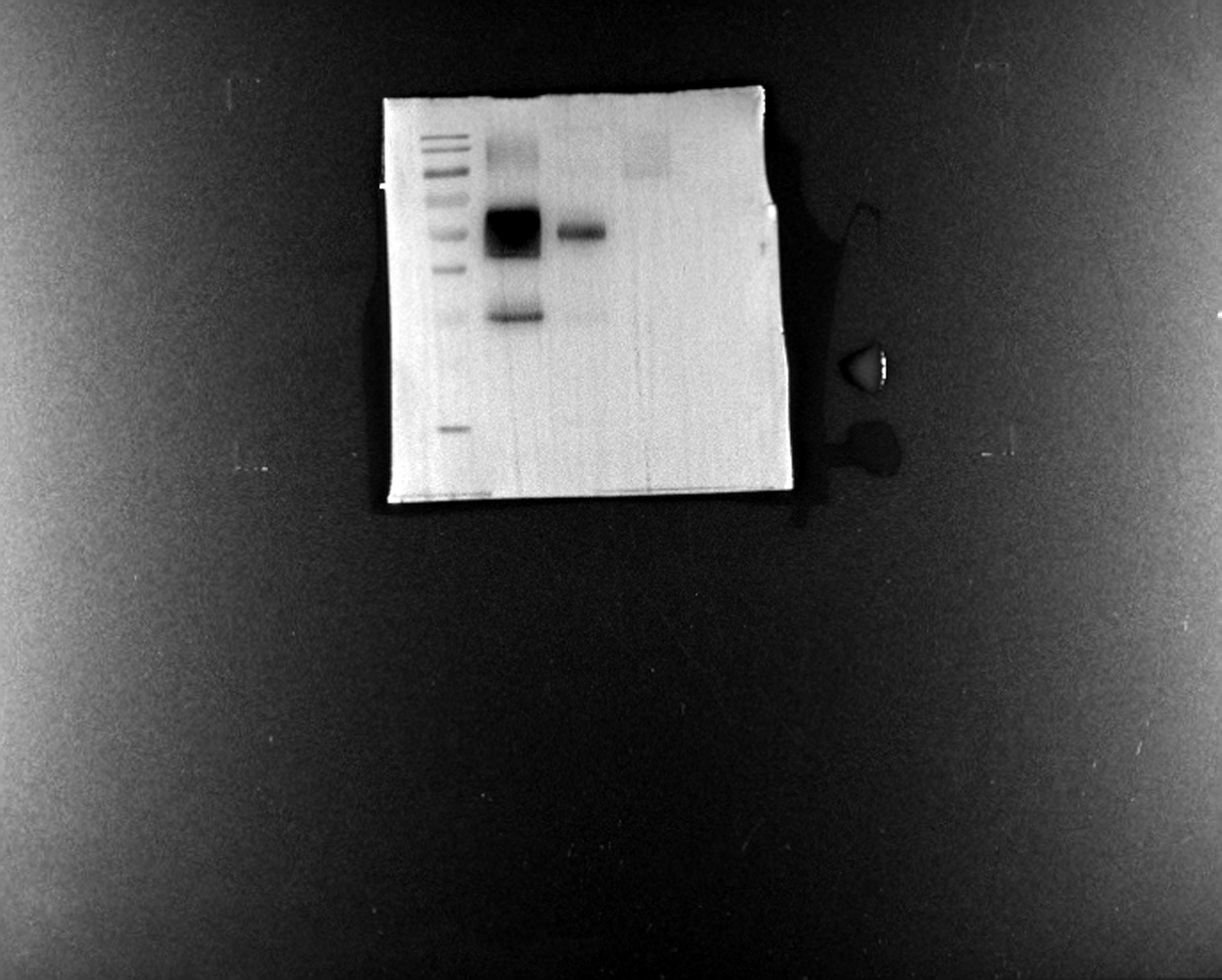

Supplement: Supplementary file 1 [file Image1.tif]
