# Supplementary material for: Two major human phenotypes of MICA molecules and their differential activation to NK cells via NKG2D receptor
Source: Front Immunol. 2025 May 19;16:1563872. doi: 10.3389/fimmu.2025.1563872 (PMC12127157; doi:10.3389/fimmu.2025.1563872)
Supplement: Supplementary file 2 [file Table1.docx]

Supplementary Material

# Supplementary Table

**Supplementary Table 1. The distribution frequency of 29 *MICA* alleles**

|  | *MICA*  allele | Chinese  （N=144） | American  （N=103） | Japanese  （N=130） | European  （N=154） | African  （N=201） |
| --- | --- | --- | --- | --- | --- | --- |
| 29 *MICA* alleles | 001 | 0.00 | 1.50 | 0.00 | 7.86 | 1.20 |
|  | 002 | 18.06 | 15.30 | 14.60 | 16.18 | 27.90 |
|  | 004 | 0.69 | 5.50 | 9.20 | 14.99 | 18.70 |
|  | 005 | 0.00 | 0.00 | 0.00 | 0.67 | 0.00 |
|  | 007 | 2.09 | 4.00 | 1.20 | 1.24 | 0.70 |
|  | 008 | 17.01 | 37.70 | 30.80 | 25.31 | 32.40 |
|  | 009 | 7.64 | 8.60 | 16.50 | 7.13 | 5.70 |
|  | 011 | 0.00 | 2.50 | 0.00 | 0.00 | 1.20 |
|  | 012 | 4.86 | 2.50 | 12.30 | 0.00 | 1.50 |
|  | 015 | 0.00 | 0.00 | 0.00 | 2.51 | 2.70 |
|  | 016 | 0.00 | 1.00 | 0.00 | 7.13 | 0.00 |
|  | 017 | 0.35 | 2.50 | 0.00 | 1.24 | 0.50 |
|  | 018 | 0.00 | 3.50 | 0.00 | 2.51 | 2.50 |
|  | 019 | 14.93 | 0.00 | 3.50 | 0.00 | 1.00 |
|  | 030 | 0.00 | 0.00 | 0.00 | 1.24 | 0.50 |
|  | 033 | 0.00 | 0.00 | 0.00 | 1.24 | 0.00 |
|  | 041 | 0.00 | 0.00 | 0.00 | 0.00 | 2.20 |
|  | 045 | 7.64 | 0.00 | 0.00 | 0.00 | 0.00 |
|  | 046 | 0.00 | 0.00 | 0.00 | 0.67 | 0.00 |
|  | 050 | 0.00 | 0.00 | 0.00 | 0.67 | 0.00 |
|  | Total | 73.27 | 84.60 | 88.10 | 90.59 | 98.70 |
| *MICA*010* allele | 010 | 22.22 | 7.00 | 10.80 | 4.55 | 0.70 |
| Other *MICA* allele | | 4.51 | 8.40 | 1.10 | 4.86 | 0.60 |
| Total | | 100 | 100 | 100 | 100 | 100 |

Note: The distribution frequency of *MICA* alleles not listed is 0.

**Supplementary Table 2. The distribution frequency of *NKG2D* alleles in population**

| *NKG2D* allele | Number (N=178) | Allele Frequency (%) |
| --- | --- | --- |
| *NKG2D*001* | 24 | 14.04 |
| *NKG2D*002* | 132 | 74.60 |
| *NKG2D*003* | 17 | 9.55 |
| *NKG2D*004* | 5 | 2.81 |
| Total | 178 | 100 |
